# Association between left atrial function using multimodality tissue tracking from cine MRI and myocardial scar in the multi-ethnic study of atherosclerosis (MESA)

**DOI:** 10.1186/1532-429X-15-S1-P266

**Published:** 2013-01-30

**Authors:** Masamichi Imai, Bharath Ambale Venkatesh, Sanaz Samiei, Mohammadali Habibi, Anderson C Armstrong, Susan Heckbert, Sirisha Donekal, David A Bluemke, Colin O Wu, Joao A Lima

**Affiliations:** 1 Johns Hopkins University, Baltimore, MD, USA; 2 Union memorial Hospital, Baltimore, MD, USA; 3 Washington University, Washington, DC, DC, USA; 4 National Institutes of Health, Bethesda, MD, USA

## Background

Left atrial (LA) function is strongly related to left ventricular (LV) filling pressures and has shown association to cardiovascular outcomes. A recently developed speckle tracking technique can assess LA deformation using CMR cine sequences. Myocardial scar assessed by late gadolinium enhancement (LGE-CMR) relates to cardiac remodeling, but its association to LA function is unknown. We explored the relationship of LA function with the amount of myocardial scar.

## Methods

A total of 1666 participants from the MESA, age range 55-94 yrs, underwent LGE-CMR using 1.5T scanners at six field centers. Myocardial scar was visually detected, classified (ischemic/non-ischemic), and quantified using semi-automated methods in 136 participants. The amount of scar was quantified as the ratio of scar mass over total LV mass and values greater than 5% were defined as clinically significant. LA function was evaluated using multimodality tissue tracking (MTT) from SSFP 2- and 4-chamber long-axis cine CMR images in all participants with myocardial scar and in an age and gender matched control group of 136 participants without scar. LA function was assessed using peak longitudinal strain (Smax), diastolic longitudinal strain rate (SRdia), ejection fraction (LAEF), emptying fraction (LAEmF), and LA maximum volume (Vmax). Wilcoxon rank-sum test was used to evaluate differences between groups: control, scar>5%, and scar<5%. Pearson's correlation assessed the relationship between the amount of scar and LA function parameters.

## Results

From the total of 136 participants with myocardial scar (72±9 yrs, 86% male, 48% ischemic), 43 participants had scars that were clinically significant (74±8 yrs, 86% male, 98% ischemic). No significant difference was found between the groups for Vmax and LAEF. LAEmF, Smax, and SRdia were the most robust parameters comparing the groups (Table 1). Both the strain parameters were significantly different between the control and the scar>5% groups while for LAEmF the difference was marginal (p=0.05). SRdia and LAEmF were significant when the scar>5% and scar<5% groups were compared, while Smax was marginally different (p=0.06). None of the parameters differed between the control and the scar<5% groups. The percent LV scar correlated significantly with LAEmF (r=-0.21, p=<0.02), Smax (r=-0.18, p<0.04), and SRdia (r=0.18, p=0.04) but not with Vmax (p=0.08) and LAEF (p=0.20).

**Table 1:** Comparison among control group, scar<5% group, and scar>5% group with peak volume, LA ejection fraction, LA emptying fraction, peak strain, and peak diastolic strain rate. * p<0.05 for comparison between scar<5% and scar>5% groups. ^ p<0.05 for comparison between control and scar>5% groups

	Median (25th percentile ,75th percentile)		
	Control (n=136)	Scar<5% (n=93)	Scar>5% (n=43)

Peak Volume ml	75.55 (62.85, 88.11)	73.97 (63.10, 85.74)	79.37 (62.60, 102.91)

LA Ejection Fraction %	37 (29, 42)	37 (29, 42)	35 (28, 40)

LA Emptying Fraction %	48 (40, 53)	50 (42, 53)	44* (39, 50)

Peak Strain %	23.78 (19.32, 30.33)	25.31 (20.63, 31.99)	21.46^ (18.65, 26.32)

Peak Diastolic Strain Rate %/ms	-0.70 (-1.00, -0.45)	-0.80 (-1.04, -0.49)	-0.59*^ (-0.83, -0.44)

## Conclusions

LA parameters are associated with cardiac remodeling in clinically significant myocardial scar. LAEmF, Smax and SRdia are more sensitive to changes in cardiac function resulting from the presence of myocardial scar than traditional LA functional parameters Vmax and LAEF.

## Funding

NHLBI N01-HC-95159

NHLBI N01-HC-95168

NCRR UL1-RR-024156

NCRR UL1-RR-025005

